# Multimodality assessment of the coronary microvasculature with TIMI frame count versus perfusion PET highlights coronary changes characteristic of coronary microvascular disease

**DOI:** 10.3389/fcvm.2024.1395036

**Published:** 2024-06-20

**Authors:** Nicole Wayne, Qufei Wu, Stephen C. Moore, Victor A. Ferrari, Scott D. Metzler, Marie A. Guerraty

**Affiliations:** ^1^ Department of Medicine, University of Pennsylvania Perelman School of Medicine, Philadelphia, PA, United States; ^2^ Center for Clinical Epidemiology and Biostatistics, University of Pennsylvania Perelman School of Medicine, Philadelphia, PA, United States; ^3^ Department of Radiology, University of Pennsylvania Perelman School of Medicine, Philadelphia, PA, United States

**Keywords:** TIMI frame count, cardiac perfusion PET, coronary microvascular disease, myocardial blood flow (MBF), imaging

## Abstract

**Background:**

The diagnosis of coronary microvascular disease (CMVD) remains challenging. Perfusion PET-derived myocardial blood flow (MBF) reserve (MBFR) can quantify CMVD but is not widely available. Thrombolysis in Myocardial Infarction (TIMI) frame count (TFC) is an angiography-based method that has been proposed as a measure of CMVD. Here, we compare TFC and PET-derived MBF measurements to establish the role of TFC in assessing for CMVD. We use coronary modeling to elucidate the relationship between MBFR and TFC and propose TFC thresholds for identifying CMVD.

**Methods:**

In a cohort of 123 individuals (age 58 ± 12.1, 63% women, 41% Caucasian) without obstructive coronary artery disease who had undergone perfusion PET and coronary angiography for clinical indications, we compared TFC and perfusion PET parameters using Pearson correlation (PCC) and linear regression modeling. We used mathematical modeling of the coronary circulation to understand the relationship between these parameters and performed Receiver Operating Curve (ROC) analysis.

**Results:**

We found a significant negative correlation between TFC and MBFR. Sex, race and ethnicity, and nitroglycerin administration impact this relationship. Coronary modeling showed an uncoupling between TFC and flow in epicardial vessels. In ROC analysis, TFC performed well in women (AUC 0.84–0.89) and a moderately in men (AUC 0.68–0.78).

**Conclusions:**

We established an inverse relationship between TFC and PET-derived MBFR, which is affected by patient selection and procedural factors. TFC represents a measure of the volume of the epicardial coronary compartment, which is increased in patients with CMVD, and performs well in identifying women with CMVD.

## Introduction

Coronary microvascular disease (CMVD) leads to angina, myocardial infarction, and heart failure (1, 2). CMVD also worsens the prognosis in patients with coronary artery disease (CAD) and is an independent risk factor for major adverse cardiovascular events (3, 4). However, challenges in diagnosing CMVD have limited both research efforts and clinical care of individuals with suspected or confirmed CMVD. Furthermore, although the prevalence of CMVD is similar in men and in women (5), the under-diagnosis of CMVD in women contributes to the discrepant burdens of untreated heart disease between women and men (1, 2, 6, 7).

Recent advances in imaging technology have allowed researchers to study and diagnose CMVD. Positron emission tomography (PET) perfusion imaging allows for the quantification of myocardial blood flow (MBF) and MBF reserve (MBFR), the ratio of MBF during hyperemic or stress conditions and rest MBF. In the absence of obstructive epicardial coronary stenoses, MBFR reflects coronary microvascular function, or the ability of the coronary microvasculature to vasodilate appropriately. PET is the current gold standard for non-invasive diagnosis of CMVD, but its availability is limited to selected tertiary care centers (8). Studies have established PET imaging as a reliable tool for detecting coronary microvascular dysfunction, and MBFR < 2 is often used as a measure of CMVD (9–12), and predicts adverse cardiovascular outcomes in patients (13). Other imaging modalities to diagnose CMVD are actively being studied and developed, including magnetic resonance imaging, echo doppler, and computed tomography (CT)-based techniques (8, 14, 15).

Coronary-angiography-based techniques have also been developed to assess the coronary microvasculature. Thrombolysis in Myocardial Infarction (TIMI) frame count (TFC) estimates the amount of time that contrast material takes to fill the epicardial vessels during a coronary angiogram. High TFC is associated with worse prognosis in the context of myocardial infarction and has also been shown to be an independent prognostic indicator of adverse events in women with signs and symptoms of ischemia without obstructive CAD (3, 16, 17). TFC also correlates well with invasive measures of coronary flow, such as average peak velocity (15, 16, 18, 19) and is associated with clinical outcomes, such as hospitalizations for chest pain (20). Coronary Vasomotion Disorders International Study recommendations recognize Coronary Slow Flow Phenomenon (defined as corrected TFC > 27) as evidence of impaired microvascular dysfunction which can be equated to a diagnosis of CMVD (15, 19, 21). Furthermore, whereas access to perfusion PET is limited by cost and availability, TFC is available for all patients undergoing coronary angiography and provides gold-standard epicardial CAD assessment along with measures of the coronary microvasculature. However, a recent study found that in patients with angina and non-obstructed coronary arteries, corrected TFC was not diagnostic of CMVD (22). Another study evaluated the correlation between coronary flow velocity reserve and TFC in patients undergoing non-emergent percutaneous coronary intervention (PCI) and found no correlation (23). Thus, despite the potential of TFC to be used in the diagnosis of CMVD (3, 24), it has not been widely adopted.

In this study, we compare PET myocardial blood flow values to TFC to gain insight into TFC measurements. We also use coronary flow modeling to better understand the relationship between TFC and flow parameters and reconcile disparate data. We then assess the effect of sex, race and ethnicity, and nitroglycerin administration on the relationship between TFC and flow parameters. Finally, we propose TFC thresholds that may in time be useful for clinically identifying CMVD and provide important context for interpreting TFC.

## Methods

### Population

We identified 350 patients who underwent both Rubidium-82 (Rb-82) perfusion PET stress testing and coronary angiography from 2012 to 2015 at the University of Pennsylvania Health System for clinical indications as part of routine medical care. We excluded those with prior heart transplantation, obstructive CAD on angiography (defined as >50% stenosis according to performing physician), or poor image quality on perfusion PET or coronary angiogram. TFC and Perfusion PET data were collected for the remaining 123 patients (age 58 ± 12.1, 63% women).

### Consent

The study was approved by the University of Pennsylvania Institutional Review Board and no informed consent was required for this retrospective study using data from the Electronic Health Record.

### Rubidium-82 (Rb-82) cardiac PET perfusion imaging

Patients underwent ^82^Rb cardiac PET perfusion imaging under both rest and dipyridamole-induced stress conditions using a Siemens Biograph mCT PET/CT scanner. Briefly, low dose CT images were acquired for attenuation correction. Rest images were obtained with a 6 min list-mode dynamic PET acquisition imaging while 30 mCi of ^82^Rb was injected intravenously as a fast bolus. Dipyridamole (0.56 mg/kg) was then administered and dynamic PET imaging was repeated with an additional 30 mCi of ^82^Rb three minutes after the completion of the infusion. Iterative reconstruction was performed with 2 iterations and the matrix size of 128 × 128 (25, 26). Global and regional MBF and MBFR were calculated using Syngo® MBF software (27). MBF was quantified using Syngo MBF, which uses a 2-compartement model developed by Hutchins et al. (28).

### TIMI frame count (TFC)

Clinical coronary angiography images were used to measure TFC using anatomic landmarks outlined in Gibson et al. (3) Coronary angiograms were acquired during routine clinical care with a frame rate of 15 frames per second, and multiplied by 2 to calculate TFC. Since TFC is determined for each of the three coronary arteries, we included patients even when TFC was only available for two coronary arteries. A TFC was not measured in a coronary territory when image quality was poor or image availability was limited. Coronary angiograms were acquired within 12 months of PET scans. TFC was measured by an investigator blinded to PET parameters. TFC was measured using the coronary angiographic projection that best visualized proximal and distal landmarks.

### Computational coronary modeling

We implemented a modified version of the Zhou-Kassab-Molloi (ZKM) symmetric coronary-tree model, including 24 Strahler levels (or vessel sizes) with coronary branching based on anatomical observations (29) (more details of the implementation are in Supplementary Figure S1). Our implementation of this model included 24 Strahler levels (or vessel sizes), where the length and volume of each was estimated (Supplementary Figure S1A–C). The flow is computed as MBF = P/R, where P is the driving pressure (100 mmHg) and R is the tree resistance. R is calculated using Poiseuille's Law to determine the resistance of each Strahler level and combining segments of the same level in parallel and different levels in series. TFC is modeled as the time to fill the two largest Strahler levels. The model was modified from ZKM (29) by allowing the individual levels to contract and dilate from their nominal vessel diameters to determine the impact of MBF and TFC.

### Statistics

The relationship between TFC from different territories was analyzed using Pearson correlation coefficient (PCC). The relationship between TFC and MBF/MBFR was analyzed using linear regression analysis, and stratified by sex, race and ethnicity, and whether nitroglycerin was used during the procedure. Multivariant linear regression modeling was performed using SAS Version 9.4. Absolute numbers and percentages were used to describe the patient population. Continuous variables were expressed as mean ± standard deviation. *T*-test was used to compare groups, and *P* < 0.05 was considered significant. Adjusting MBF by heart rate-blood pressure product did not alter the results, and therefore the unadjusted MBF values are presented. Receiver operating curves (ROC) were generated and fitted using the maximum-likelihood approach (30) implemented in the program LABROC, which is available as part of the Metz ROC Software (University of Chicago). The ROC data were fitted using a semi-parametric method, “proper” binormal model (31), and the inverse information matrix was used to estimate parameter uncertainties. Area Under the Curve (AUC) is presented and *P* < 0.05 was considered significant.

## Results

We identified a cohort of 123 patients (age 58 ± 12.1, 63% women) who were eligible for this study (Table 1). This population was racially and ethnically diverse (54% black) with high prevalence of cardiovascular risk factors. There were a similar number of patients with and without CMVD. CMVD was defined as MBFR <2 on perfusion PET stress test.

**Table 1:** Population demographics.

Characteristic	Total: *N* = 124 (%)	Men: *N* = 46 (%)	Women: *N* = 78 (%)
Age	58 ± 12.1	58 ± 12.5	59 ± 12.0
Race			
Asian	3 (2.42%)	1 (2.2%)	2 (2.6%)
Hispanic	3 (2.42%)	0 (0%)	3 (3.8%)
Black	67 (54.03%)	23 (50%)	44 (56.4%)
White	51 (41.13%)	27 (58.7%)	24 (30.8%)
BMI (mean ± SD)	38.1 ± 9.7	36.3 ± 9.7	39.1 ± 9.8
Hyperlipidemia	97 (78.23%)	36 (78.3%)	60 (76.9%)
Hypertension	115 (92.74%)	39 (84.8%)	76 (97.4%)
Diabetes Mellitus	57 (45.97%)	20 (43.5%)	37 (47.4%)
Tobacco use (ever)	36 (29.03%)	14 (30.4%)	22 (28.2%)
Abnormal global MBFR (defined as MBFR < 2)	69 (55.6%)	24 (52.2%)	45 (57.7%)
LAD mean TFC (mean ± SD)	36 ± 9.1	39.4 ± 11.6	33.9 ± 6.6
LCX mean TFC (mean ± SD)	34 ± 7.5	35.3 ± 9.0	32.9 ± 6.5
RCA mean TFC (mean ± SD)	36 ± 8.2	38.3 ± 10.2	34.4 ± 6.5

**Figure 1:**
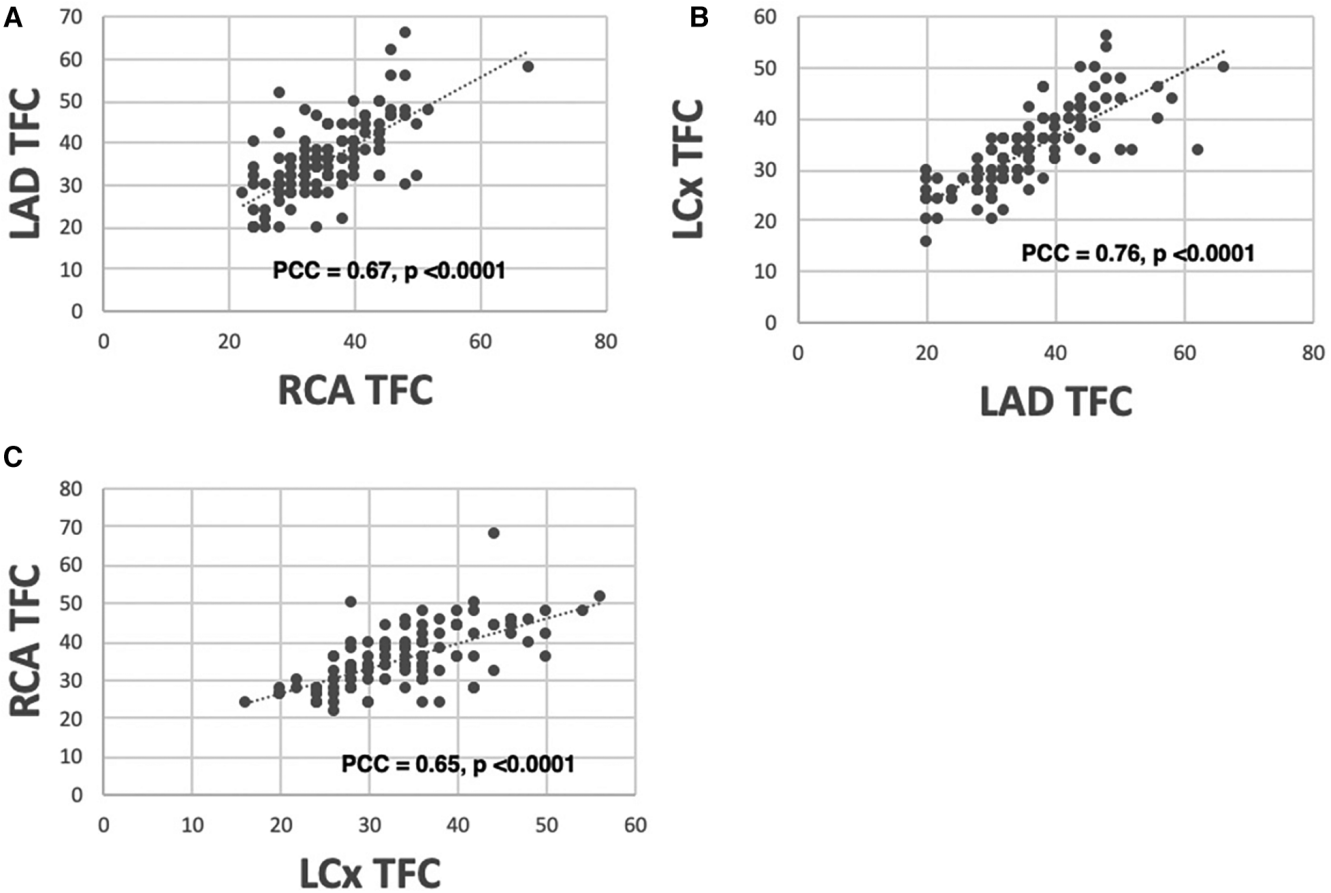
Correlation between TFC measured from different coronary territories. There is a high correlation between TFC obtained from LAD vs. RCA [(**A**) PCC = 0.67], LAD vs. LCX [(**B**) PCC = 0.76], and RCA vs. LCX [(**C**) PCC = 0.65].

Because patients with CAD were excluded, there was a strong positive correlation between the global MBFR and regional MBFR for each coronary territory, with PCC 0.91, 0.96, and 0.97 for the right coronary artery (RCA), left circumflex artery (LCX) and left anterior descending artery (LAD), respectively. There was a positive, although weaker, correlation in TFC between different regions (LAD vs. LCx: PCC = 0.76; LAD vs. RCA: PCC = 0.67; LCx vs. RCA: PCC = 0.65) (*p* < 0.0001 for all territories) (Figure 1).

**Figure 2:**
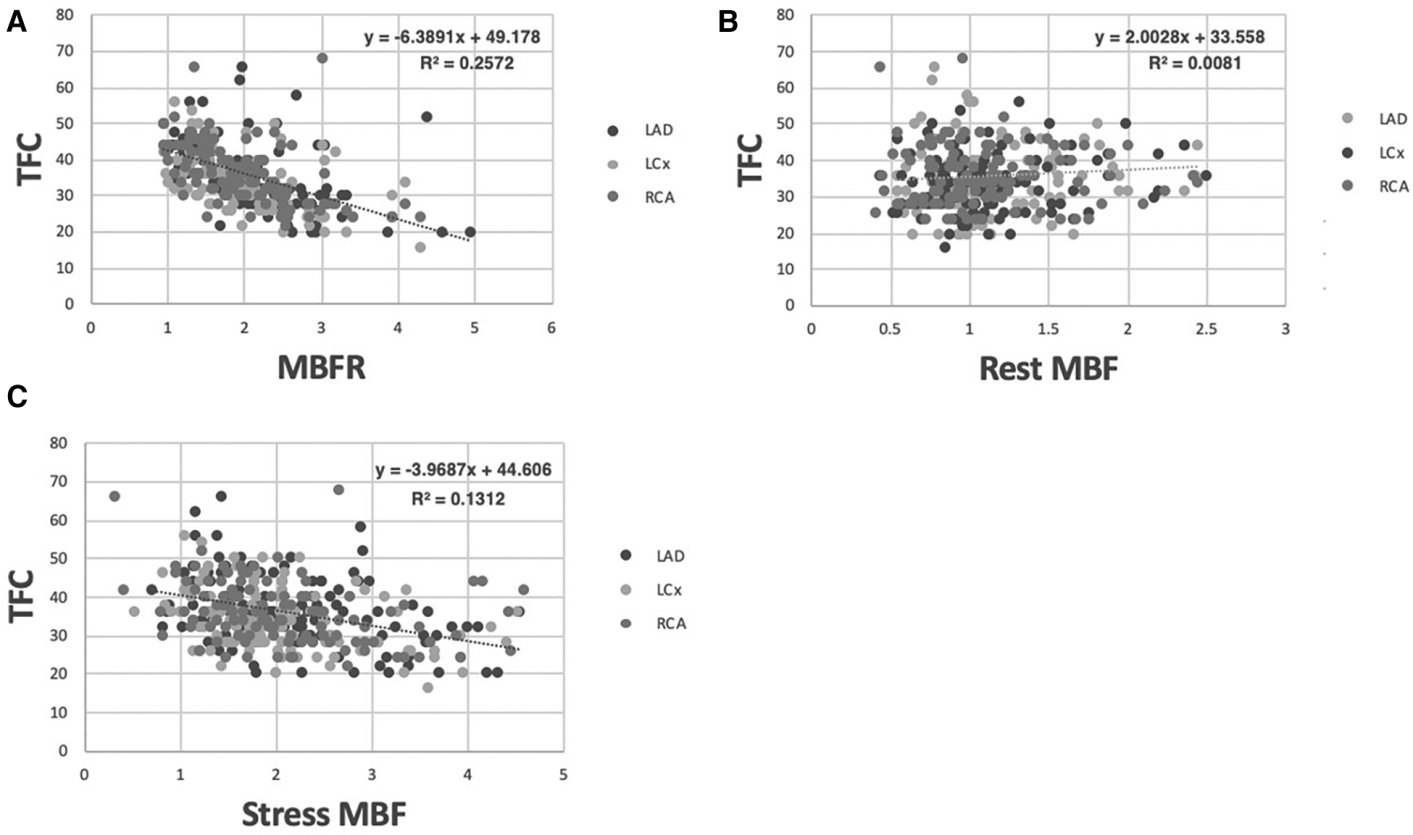
Correlation between TFC and MBFR, rest MBF, and stress MBF in all patients. (**A**) Correlation between MBFR and TFC in all patients (*p* < 0.0001 for all 3 coronary territories). (**B**) Resting myocardial blood flow vs. TFC across coronary territories in all patients lacks a significant relationship. (**C**) Stress myocardial blood flow vs. TFC across coronary territories in all patients shows a significant but only slight inverse relationship.

We next compared the relationship between TFC for each coronary artery and corresponding regional MBFR. We found an inverse relationship with PCC −0.51, −0.54, and −0.51 for RCA, LCX, and LAD, respectively (Figure 2A, *p* < 0.0001 for all). We next examined the two components that comprise MBFR: rest MBF and stress MBF. There was no statistically significant correlation between TFC and rest MBF in all patients, but there was a trend towards increasing TFC with higher rest MBF in LAD and LCX. PCC between TFC and rest MBF were 0.127 (*p* = 0.17), −0.04 (*p* = 0.67), and 0.108 (*p* = 0.24) for LAD, RCA, and LCX, respectively (Figure 2B). There was a slight negative relationship between TFC and stress flows with correlations of −0.364, −0.512, −0.384 in LAD, RCA, and LCX, respectively (Figure 2C, *p* < 0.0001 for all).

The strong inverse relationship between MBFR and TFC was counterintuitive. TFC is a measure of how much time is required for epicardial coronary arteries to fill with contrast and has been linked to coronary flow measurements. Depending on the dose and type of moderate sedation used during the coronary angiogram, this may represent a rest or stress (hyperemic) state. However, we found a stronger association with MBFR, which is a unitless ratio and reflects the ability of the coronary microvasculature to vasodilate. To better understand the association between MBFR and TFC, and to reconcile disparate results with prior published work (22), we turned to coronary flow modeling.

**Figure 3:**
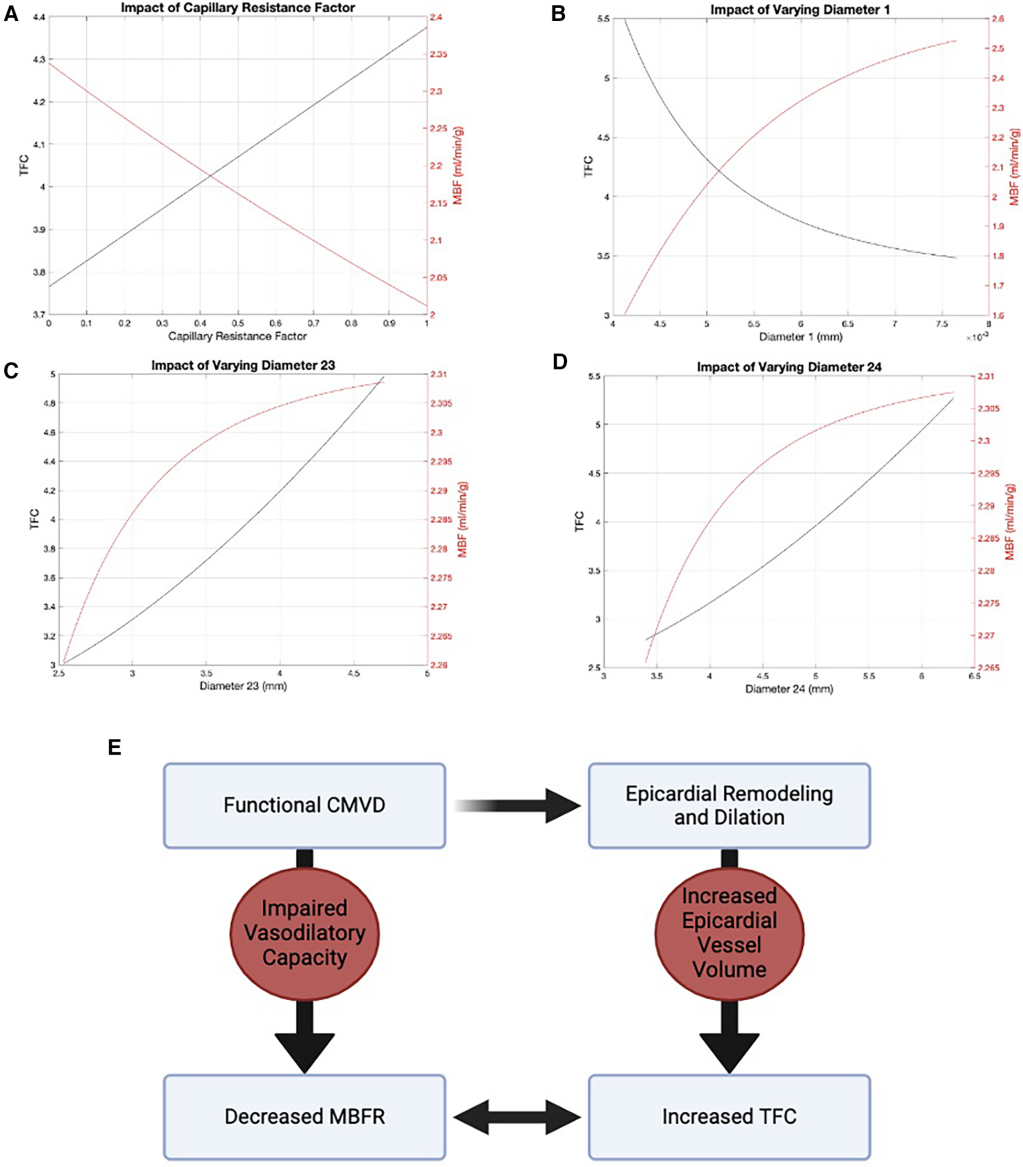
Modified ZKM model of the coronary circulation. There is an inverse relationship between flow (red) and TFC (black) in the two smallest vessel levels: (**A**) level 0 and (**B**) level 1. In the two largest vessels, there is an uncoupling of flow and TFC: (**C**) level 23 and (**D**) level 24. (**E**) Diagram demonstrating the relationship between MBFR and TFC via epicardial remodeling.

We used a modified Zhou-Kassab-Molloi (ZKM) model (29) of the coronary circulation to investigate the association between MBFR and TFC. Figure 3 shows how TFC and flow varied as the diameters of the two largest and two smallest vessel levels are varied, with all other levels unchanged. In the largest vessels, there was minimal change in flow over a large range of diameters (Figures 3C,D), consistent with known coronary physiology where the large epicardial vessels are not the primary regulators of coronary flow. When looking at all 24 Strahler levels (or coronary vessel diameters), we found that TFC and blood flow were inversely correlated at all levels except the two largest diameter vessel compartments (Supplementary Figure S2). These are the epicardial coronaries visualized by coronary angiography and in which TFC is measured. Coronary modeling shows an uncoupling of blood flow and TFC in these largest vessels, suggesting TFC is predominantly affected by the volume of the epicardial coronary compartment rather than the flow rate. Thus, CMVD may result in larger proximal vessels due to remodeling, so that even though the flow rate is larger at rest (as measured by MBF), the clearance of contrast from the epicardial vessels (as measured by TFC) is decreased (Figure 3E).

**Figure 4:**
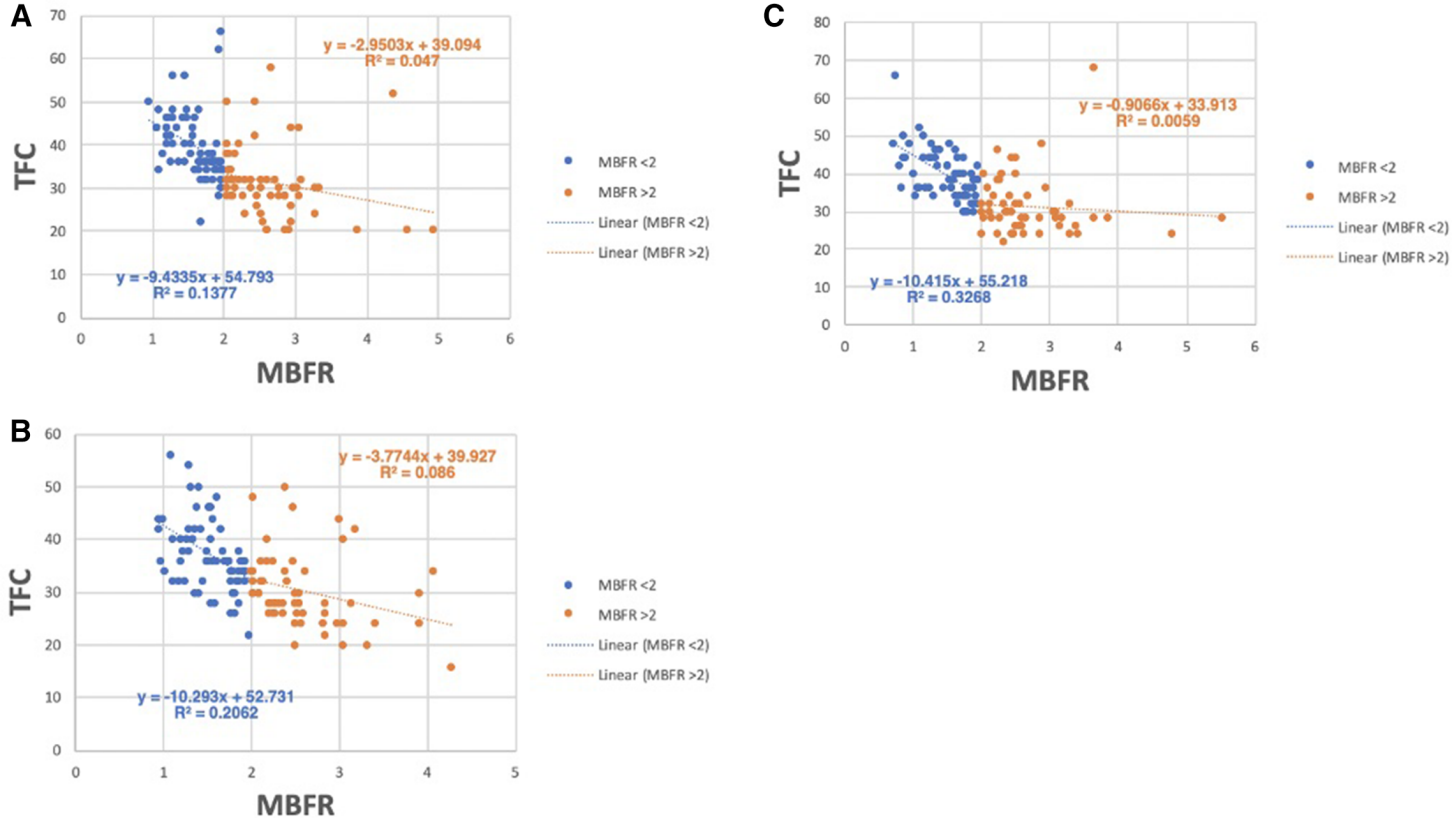
Relationship between TFC and MBFR in patients with and without CMVD (MBFR ≥ 2). (**A**–**C**) TFC vs. MBFR for participants with (MBFR < 2) and without CMVD in the (**A**) LAD, (**B**) LCx, and (**C**) RCA, respectively, highlights the strong relationship between TFC and MBFR present in those with CMVD (blue) and a more blunted slope in patients without CMVD (orange).

Based on this model, we expect the relationship between TFC and MBFR to be stronger in patients with CMVD (MBFR <2). We therefore determined whether the degree of CMVD (defined as MBFR < 2) affected the correlation between MBFR and TFC. In all coronary territories, the correlation between MBFR and TFC was stronger in those with MBFR < 2 (LAD PCC −0.371 vs. −0.217, LCx PCC −0.454 vs. −0.293, RCA PCC −0.572 vs. −0.077; *p* < 0.0001 for MBFR < 2 vs. ≥2, respectively) (Figures 4A–C). This result gives insight into discrepant results between studies, since the relationship between TFC and MBFR depends on the prevalence of cardiovascular risk factors and CMVD (MBFR < 2) in the study population.

Since modeling showed that TFC is predominantly a measure of the volume of the epicardial compartment, medications that directly vasodilate the proximal coronaries, such as nitroglycerin (NTG), may affect TFC measurements. Therefore, we analyzed whether the relationship between MBFR and TFC was reduced for patients who had received NTG during coronary angiography. At our institution before 2010, patients underwent cardiac catheterization via femoral access and the vast majority of these patients did not receive NTG. Between 2010 and 2015, the institution transitioned from femoral to radial access and NTG was commonly used. Overall, 31 patients in this cohort (30% of men and 22% of women) received NTG. We re-evaluated the association between TFC and MBFR in each coronary territory, stratified by administration of NTG. In each coronary vessel, we found that the correlation between TFC and MBFR was stronger in patients who did not receive NTG than in those who did (NTG vs. no NTG, respectively: LAD PCC −0.211 vs. −0.513, LCx PCC −0.197 vs. −0.546, RCA PCC −0.234 vs. −0.605, *p* < 0.0001 for all). To ensure that these differences were not due to differences in sample sizes, we repeated the analysis with three randomly selected, down-sampled populations. The results were unchanged.

**Figure 5:**
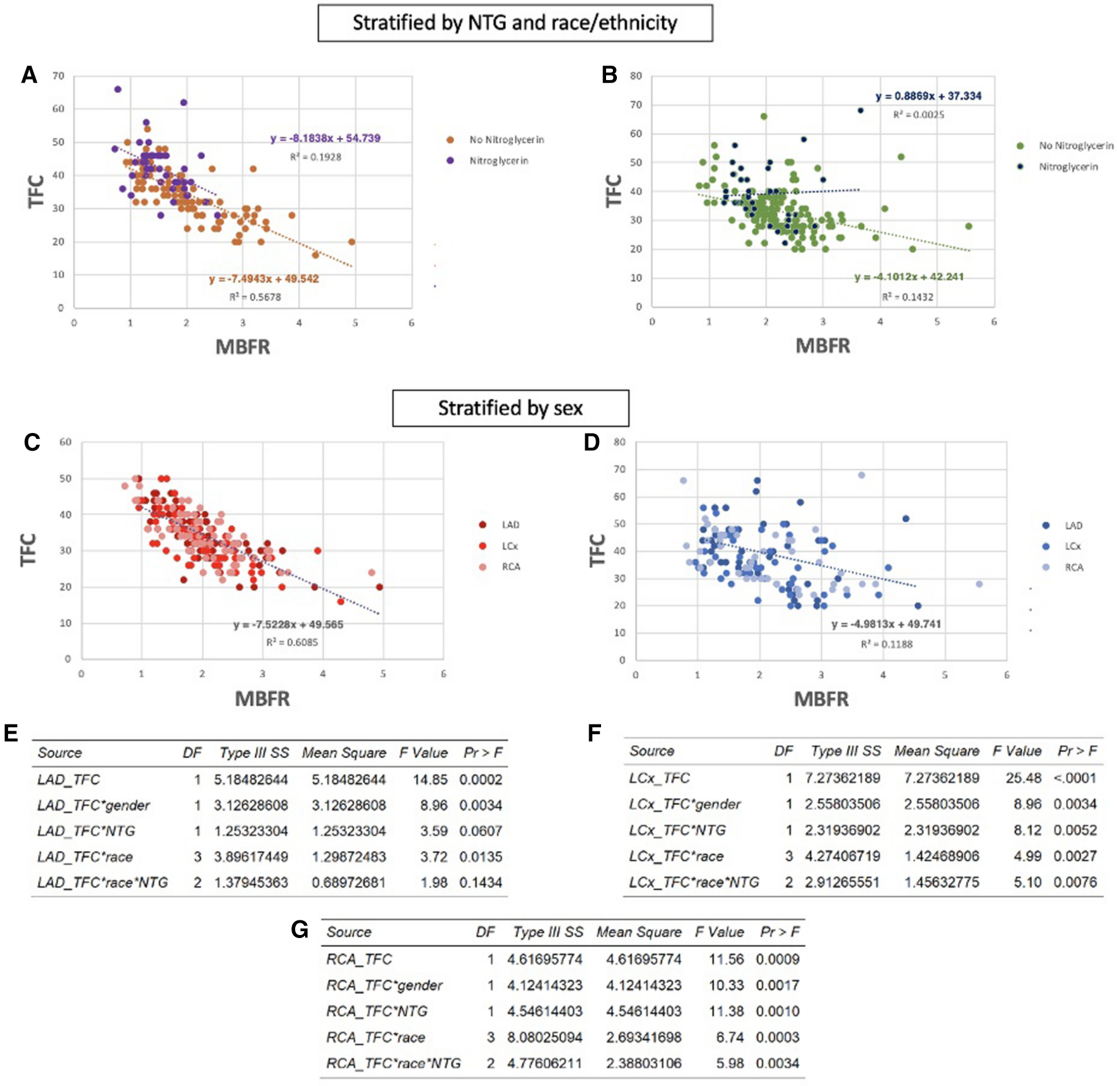
Nitroglycerin (NTG) administration, race and ethnicity, and sex impact the relationship between TFC and MBFR. (**A,B**) Correlation between MBFR and TFC in all coronary territories in white patients (**A**) and black patients (**B**) who received nitroglycerin versus those who did not. The inverse relationship between TFC and MBFR is eliminated in Black patients who received NTG. (**C**,**D**) The association between TFC and MBFR was much stronger in women (**C**) compared to men (**D**) (**E**–**G**) Multivariant linear regression modeling highlights the significant effect of these confounding variables on the relationship between TFC and MBFR in the LAD (**E**), LCx (**F**), and RCA (**G**) territories.

Race and ethnicity are known to affect response to NTG (32). We therefore assessed whether race and ethnicity impacted the effect of NTG on the relationship between MBFR and TFC. We found a moderate correlation between TFC and MBFR in white individuals who did and did not received NTG (PCC −0.439 and −0.753, respectively, *p* < 0.0001) (Figure 5A). However, in black individuals, there was no correlation between TFC and MBFR in those who received NTG (PCC 0.050, and only a mild correlation in those who did not PCC −0.378, *P* < 0.0001) (Figure 5B).

Prior studies have identified sex-based differences in remodeling (33). Thus, we looked at the effect of sex on the relationship between MBFR and TFC. Interestingly, there was a strong sex difference in the negative correlation between TFC and regional MBFR. In women, correlations between TFC and MBFR were −0.73, −0.74, and −0.78 for RCA, LCX, and LAD, respectively (Figure 5C, *p* < 0.0001 for all). The relationship between TFC and regional MBFR also existed in men but was weaker, with PCC −0.41, −0.38, and −0.35 for RCA, LCX, and LAD, respectively (Figure 5D, *p* < 0.0001 for all). As above, we repeated the analysis with three randomly down-sampled populations, and the results remained consistent. To formally establish whether these confounding factors interacted with TFC, we used multivariate linear regression analysis including sex, race and ethnicity, and nitroglycerin use. We found that all of these variables have a significant impact on the relationship between TFC and MBFR (Figures 5E–G).

**Figure 6:**
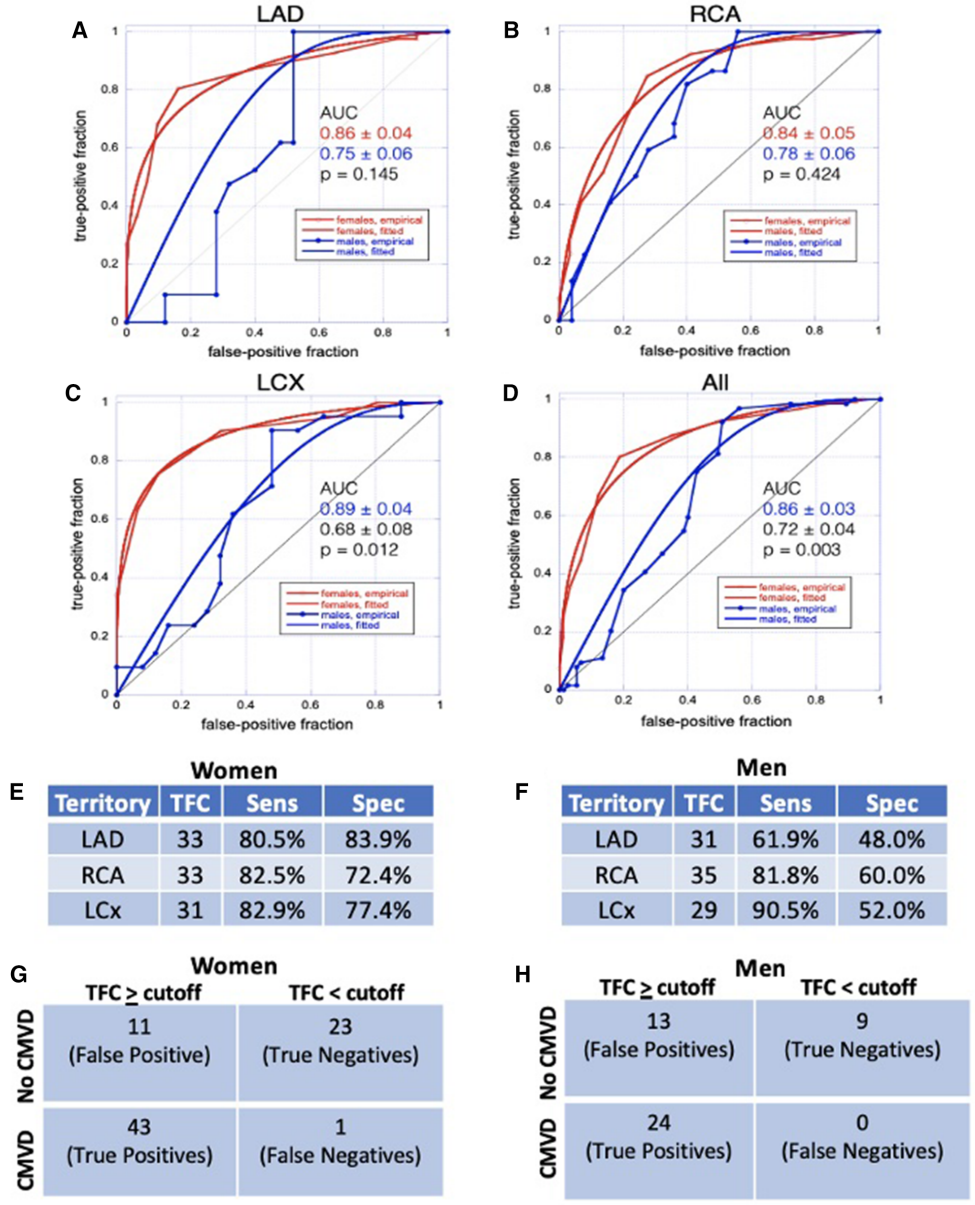
ROC analysis. (**A**–**D**) ROC analyses for TFC in LAD (**A**), RCA (**B**), LCX (**C**), and all territories combined (**D**) show that TFC performs better in women than in men. (**E**, **F**) Sensitivity and specificity for TFC values chosen from ROC analyses as diagnostic thresholds for CMVD in women (**E**) and in men (**F**) (**G**,**H**) Contingency table demonstrating true positives, true negatives, false positives, and false negatives for women (**G**) and men (**H**); CMVD is defined as MBFR < 2 and TFC ≥ cutoff is defined as TFC above defined threshold (**E**,**F**) in one or more coronary territory.

Lastly, we sought to assess the ability of TFC to identify patients with CMVD, defined as MBFR < 2 (13). We performed receiver operating characteristic (ROC) analysis for each territory separately and all together, stratified by sex (Figures 6A–D). TFC showed excellent performance in identifying CMVD in women with some slight variability among territories (AUC = 0.86, 0.84, and 0.89 for LAD, RCA and LCX, respectively). TFC performed less well in men in all territories (AUC = 0.75, 0.78, and 0.68 for LAD, RCA and LCX, respectively). In women, TFC thresholds for identifying CMVD were 33 for the LAD (sensitivity 80.5%, specificity 83.9%), 33 for the RCA (sensitivity 82.5%, specificity 72.4%), and 31 for the LCx (sensitivity 82.9%, specificity 77.4%). In men, TFC thresholds for identifying CMVD were 31 for the LAD (sensitivity 61.9%, specificity 48%), 35 for the RCA (sensitivity 81.8%, specificity 60%), and 29 for the LCx (sensitivity 90.5%, specificity 52%) (Figures 6E–H). When all territories were considered, there was a statistically significant difference in TFC performance between women and men (AUC = 0.86 vs. 0.72, *p* = 0.003). Based on ROC curves, we established TFC thresholds that identify CMVD with sensitivity > 80% in women for all three coronary arteries (Figures 6E,G). The high territory-specific variability limited the sensitivity in men (Figures 6F,H).

## Discussion

In a diverse cohort of patients, we demonstrated a relationship between TFC from clinical coronary angiograms and perfusion PET MBFR. Using computational modeling, we show how the relationship between coronary flow and TFC breaks down in the larger epicardial vessels that are used for TFC measurement. These data support a model where TFC is predominantly driven by epicardial coronary volume and reflects underlying disease rather than a physiologic parameter. We also identified several variables that confound the relationship between TFC and MBFR including sex, race and ethnicity, and nitroglycerin administration. Finally, we show that TFC has potential diagnostic utility in CMVD, especially in women.

We identified a strong relationship between TFC and MBFR, which was not driven by rest or stress MBF. TFC is thought to be primarily driven by coronary flow (18) and TFC correlated with coronary flow velocity under adenosine-induced hyperemic conditions in a cohort of 11 patients (34). In our study, there was a minimal relationship between rest MBF and TFC and a slight inverse relationship between TFC and stress MBF. Coronary modeling helps to explain these disparate data. We show that there is an uncoupling between flow and TFC in larger vessels, which allows for increased TFC in the presence of increased resting flow and/or decreased flow reserve. This indicates that TFC is predominantly reflective of the volume of the epicardial compartment, rather than the flow rate in the epicardial coronary artery.

CMVD is associated with distal endothelial dysfunction leading to impaired vasodilatory response to both pharmacological and physiological stimuli (2, 35). Some studies suggest CMVD is associated with vascular remodeling (33, 36). A recent study also showed that patients with functional CMVD have increased proximal epicardial vessel volume, suggestive of vascular remodeling in response to more distal microvascular dysfunction (37). These studies point to proximal remodeling in response to microvascular dysfunction with increased epicardial vessel volume, consistent with our findings. In summary, CMVD leads to epicardial remodeling and changes in epicardial vessel volume, which results in increased TFC (time to fill the vessel).

In support of this theory, we find that the relationship between TFC and MBFR is stronger in patients with CMVD (MBFR < 2), who are more likely to have remodeling. Additionally, the relationship between TFC and MBFR is diminished in patients who received nitroglycerin prior to catheterization. This would serve to artificially dilate the proximal coronary arteries in those without CMVD (MBFR > 2), artificially increase TFC, and obscure the difference between normal individuals and those with CMVD.

In summary, TFC serves as a measure of disease in individuals rather than a pure measure of coronary blood flow. At the patient level, this result is consistent. TFC and MBFR are both measures of CMVD, and patients with more severe CMVD have both lower MBFR and higher TFC. This is further supported by the fact that PET and coronary angiography were performed within days-months of each other but not at the same time, suggesting that this reflects underlying pathophysiology instead of a snapshot of hemodynamic parameters.

Prior work by Gibson et al. has advocated for the use of TFC for assessing the coronary microvasculature in patients undergoing coronary angiography (3, 24). Petersen et al. have shown that resting TFC independently predicts rates of hospitalization for angina in women with signs of ischemia without obstructive CAD (16). Our data support that these two groups of patients may have CMVD as the link between increased TFC and adverse outcomes. Our results further support the use of TFC as a measure of the coronary microvasculature by establishing the feasibility of quantifying TFC from clinical angiograms rather than angiograms performed as part of research protocols.

Recent studies have reported conflicting results. Dutta et al. measured CMVD using coronary pressure and flow velocity before and after adenosine administration and concluded that Coronary Slow Flow Phenomenon (defined as corrected TFC > 27) was a poor indicator of CMVD (22). However, individuals in this study represented a generally healthier population, with higher coronary flow reserve and lower rates of hypertension, diabetes, and hypercholesterolemia, and also received NTG. Both of these weaken the relationship between TFC and flow reserve measurements.

The underlying mechanism driving the sex-difference in the relationship between TFC and MBFR is unclear. Our study cohort is enriched for women, similar to prior studies that have found larger proportions of women with symptoms and non-obstructive coronaries (38). However the weaker relationship between TFC and MBFR in men appears to be at least partially due to greater variance in TFC rather than smaller sample size (Figures 5B). Additionally, in this cohort of patients, a greater percentage of men received nitroglycerin than women, which could contribute to the stronger correlation we see in women. We tested the hypothesis that having larger epicardial coronaries impacts the relationship between TFC and MBFR in coronary modeling by increasing the Strahler levels, but saw no change in the model's behavior to explain the finding. Prior studies have identified sex-based differences in plaque formation and deposition and vascular remodeling (33). Specifically, the Women's Ischemia Syndrome Evaluation (WISE) intravascular ultrasound (IVUS) study showed that in women with symptoms of ischemia but without luminal obstruction on catheterization, there was a high prevalence of positive remodeling which maintained the coronary lumen via compensatory enlargement secondary to external elastic membrane expansion (33). It is possible than men and women have differential outward remodeling, which may differentially change the epicardial volumes and thus the TFC. Lastly, non-obstructive atherosclerosis in men may also have subtle effects on flow and lead to greater variability in TFC and MBFR measurements.

This study has several limitations. First, it is a single-center, retrospective study, with a small population of men. Second, this study links two distinct imaging biomarkers, and additional work is needed to link the heterogenous pathophysiology of CMVD with these biomarkers. Third, we use clinically-acquired coronary angiograms. These prioritize clinical care and patient safety and thus use less radiation, fewer frames, and are of poorer image quality. Additionally, each institution has its own coronary catheterization protocols, including use of NTG and other vasodilators, which can affect TFC measurements.

Future studies are needed to understand differences due to sex and race and ethnicity, to establish TFC as a diagnostic test for CMVD, and to link TFC with adverse cardiovascular outcomes in a large population without obstructive CAD. TFC has great potential as an imaging biomarker to accelerate the research into CMVD pathogenesis and may be helpful for screening patients for CMVD trials. TFC may also be a useful imaging biomarker for phenotyping CMVD in large cohorts and biobanks, such as the PennMedicine Biobank, the UK Biobank, and the Veterans Administration Million Veterans Program. However, we propose that the greatest immediate impact of this work is to encourage additional studies into the use of TFC as a simple, cost-effective, and widely available tool that can be applied to clinical coronary angiograms to assess for CMVD, especially in women with cardiac symptoms who may otherwise be falsely reassured by non-obstructive coronary arteries.

## Data Availability

The raw data supporting the conclusions of this article will be made available by the authors, without undue reservation.
