# Supplementary figures and images for: Multimodality assessment of the coronary microvasculature with TIMI frame count versus perfusion PET highlights coronary changes characteristic of coronary microvascular disease
Source: Front Cardiovasc Med. 2024 Jun 20;11:1395036. doi: 10.3389/fcvm.2024.1395036 (PMC11222597; doi:10.3389/fcvm.2024.1395036)

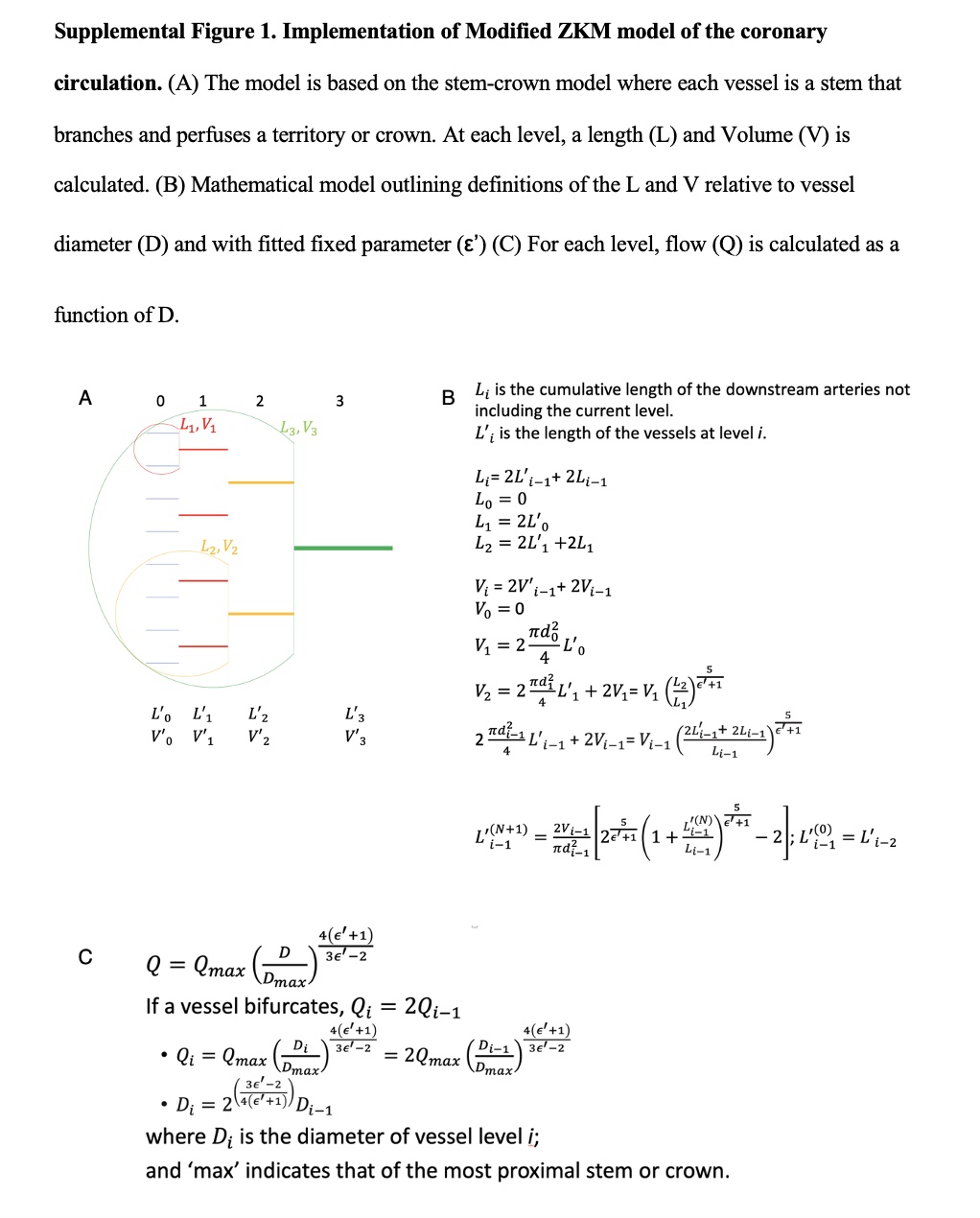

Supplement: Supplementary file 1 [file Image1.jpg]

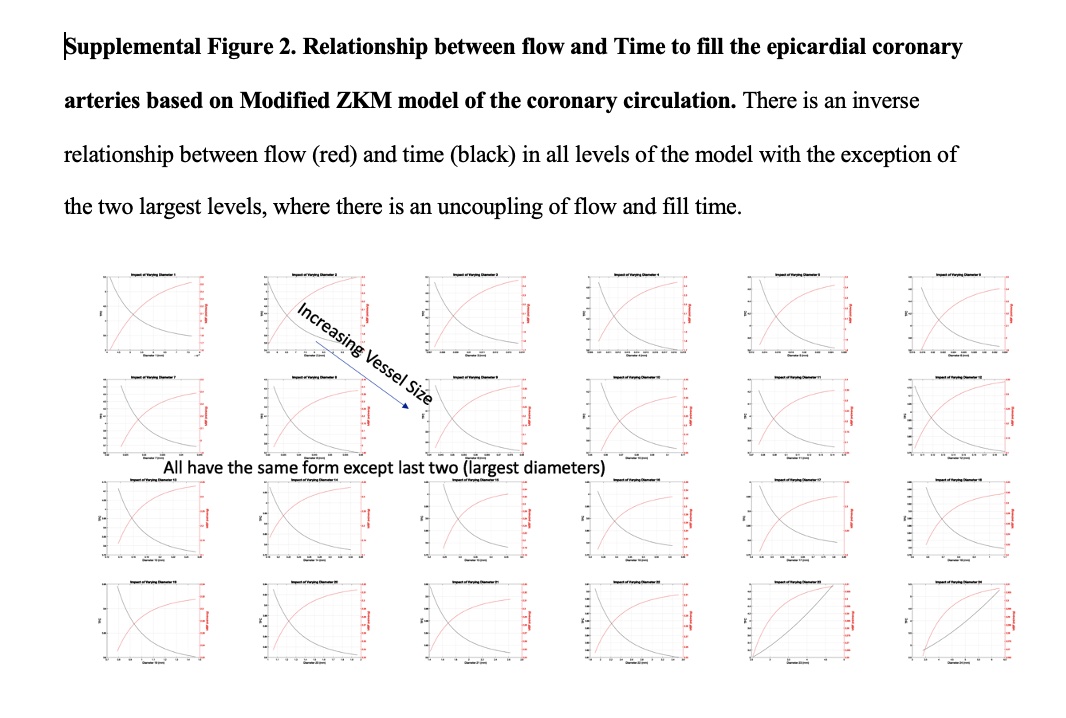

Supplement: Supplementary file 2 [file Image2.jpg]
